# Expanding the scope beyond mortality: burden and missed opportunities in maternal morbidity in Indonesia

**DOI:** 10.1080/16549716.2017.1339534

**Published:** 2017-06-26

**Authors:** Vitri Widyaningsih, Khotijah, Balgis

**Affiliations:** ^a^ Faculty of Medicine, Universitas Sebelas Maret, Surakarta, Indonesia

**Keywords:** Maternal morbidity, missed opportunity, maternal health, determinants, sociodemographic

## Abstract

**Background:** Indonesia still faces challenges in maternal health. Specifically, the lack of information on community-level maternal morbidity. The relatively high maternal healthcare non-utilization in Indonesia intensifies this problem.

**Objective:** To describe the burden of community-level maternal morbidity in Indonesia. Additionally, to evaluate the extent and determinants of missed opportunities in women with maternal morbidity.

**Methods:** We used three cross-sectional surveys (Indonesian Demographic and Health Survey, IDHS 2002, 2007 and 2012). Crude and adjusted proportions of maternal morbidity burden were estimated from 43,782 women. We analyzed missed opportunities in women who experienced maternal morbidity during their last birth (*n* = 19,556). Multilevel mixed-effects logistic regressions were used to evaluate the determinants of non-utilization in IDHS 2012 (*n* = 6762).

**Results:** There were significant increases in the crude and adjusted proportion of maternal morbidity from IDHS 2002 to IDHS 2012 (*p* < 0.05). In 2012, the crude proportion of maternal morbidity was 53.7%, with adjusted predicted probability of 51.4%. More than 90% of these morbidities happened during labor. There were significant decreases in non-utilization of maternal healthcare among women with morbidity. In 2012, 20.0% of these women did not receive World Health Organization (WHO) standard antenatal care. In addition, 7.1% did not have a skilled provider at birth, and 25.0% delivered outside of health facilities. Higher proportions of non-utilization happened in women who were younger, multiparous, of low socioeconomic status (SES), and living in less-developed areas. In multilevel analyses, missed opportunities in healthcare utilization were strongly related to low SES and low-resource areas in Indonesia.

**Conclusion:** The prevalence of maternal morbidity in Indonesia is relatively high, especially during labor. This condition is amplified by the concerning missed opportunities in maternal healthcare. Efforts are needed to identify risk factors for maternal morbidity, as well as increasing healthcare coverage for the vulnerable population.

## Background

Maternal health remains a major public health concern, particularly in developing countries. These developing countries contribute over 99% of maternal deaths in the world [1]. Globally, Indonesia ranks fifth in the largest number of maternal deaths. Moreover, Indonesia has one of the highest Maternal Mortality Ratio (MMR) in Southeast Asia [2,3]. In 1989, the Indonesian government launched the Safe Motherhood Initiative and implemented a variety of programs to improve maternal health [4–6]. These efforts have decreased the MMR from over 400/100,000 live births in the 1990s to 220/100,000 live births in 2010. This reduction rate, however, is slower compared to the Millennium Development Goals (MDGs) target of 75% reduction by 2015 [7].

To date, maternal mortality has been the target of interventions in maternal health, with less focus on maternal morbidity [8]. Maternal morbidity is defined as 'any health condition attributed to and/or complicating pregnancy and childbirth that has a negative impact on a woman’s well-being and/or functioning' [9]. Although morbidity and mortality are closely related, reports have suggested the importance of extending the programs to include prevention and reduction of maternal morbidity. It is estimated that for each maternal mortality, there are 20–30 women with acute or chronic morbidity, which often leads to disability [10]. The disability can affect not only the women but also her family and the newborn. The additional social and economic cost of the disability signifies the importance of addressing this problem [11,12]. Therefore, there is an urgent need to direct attention on maternal morbidity and improve women’s experience during pregnancy, delivery, and postpartum.

In Indonesia, the burden from maternal morbidity is amplified by the relatively high non-utilization of maternal healthcare. In 2012, 12.2% of Indonesian women did not receive the standard World Health Organization (WHO) antenatal care (ANC) visits. Additionally, 16.9% of deliveries were not attended by a skilled provider, and 36.8% of births happened outside a health facility [13]. Previous studies on maternal morbidity in Indonesia were hospital-based. Therefore, they provided limited understanding for effective public health interventions [14,15]. To design effective population intervention, information on the community-level prevalence of maternal morbidity and intervention target are crucial. This study aims to describe the burden of maternal morbidity in Indonesia. Concurrently, we also aim to demonstrate the extent and determinants of healthcare non-utilization among women with maternal morbidity. Thus, this study provides critical insights into the missed opportunities in maternal health, which can be used as the focus for more relevant public health interventions.

## Methods

### Study design

This study used three waves of Indonesia Demographic and Health Survey (IDHS) data which included maternal morbidity questions: the IDHS 2002, 2007 and 2012. These repeated cross-sectional surveys with multistage sampling designs were conducted to obtain nationally representative data.

### Setting and participants

Indonesia is the fourth most populated country in the world, with over 260 million people inhabiting almost 40% of its over 17,000 islands [16]. In this study, we classified the geographical location of Indonesia into three regions: Java–Bali, more-developed other islands and less-developed other islands. These categories were based on IDHS classifications [17].

The IDHS collected data from women who had at least one birth in five years preceding the three IDHS waves from 34 provinces in Indonesia. Thus, our sample consisted of women who gave birth during 1998–2012 (*n* = 43,782). However, approximately 3264 women (6.6%) had missing data on maternal morbidity and were excluded from the analyses. Data on healthcare utilization were only available for last (index) birth. Therefore, for analyses on missed opportunity, we only included women who experienced maternal morbidity during their last birth (*n* = 19,556, weighted *n* = 19,227). There were relatively low missing data on ANC (*n* missing = 1252, 6.4%), skilled birth attendance (SBA) (*n* missing = 67, 3.4%), and facility birth (FB) (*n* missing = 8, 0.04%). Analyses on determinants of non-utilization were conducted on IDHS 2012 (*n* = 6762, weighted *n* = 7301).

### Variables and measurements

#### Maternal morbidity

Morbidity status was obtained from the interview-based questionnaires. Women were asked if they experienced prematurity, bleeding, fever or infection, convulsion and/or other morbidities during pregnancy. For labor morbidity, the IDHS recorded self-reported data for prolonged labor, bleeding, fever or foul vaginal discharge, convulsions, and/or other morbidities. Starting in IDHS 2007, premature rupture of membrane (PROM) was also included in the labor morbidity questions. The maternal morbidity variable was constructed from a combination of labor and pregnancy morbidity.

#### Missed opportunity

To assess the missed opportunities in maternal morbidity, we evaluated three important indicators of maternal healthcare: WHO standard ANC, skilled birth attendance (SBA) and facility birth (FB). We used WHO standard ANC that requires a minimum of four antenatal visits: once during the first trimester, once during the second trimester, and twice during the third trimester.

#### Covariates

For individual-level variables, we evaluated sociodemographic variables associated with healthcare non-utilization in IDHS 2012. We included age, parity, family wealth, women’s level of education and health insurance membership as the determinants. These variables were obtained from self-reported questionnaires.

We use the IDHS 2012 sampling strata to define community. In IDHS 2012, there were 65 sampling strata representing the 33 provinces in Indonesia. Among the 33 provinces, 32 were further classified into urban and rural areas (64 strata), except DKI Jakarta which is an urban-only area [17]. At the community level, we assessed the influence of indicators that might reflect geographic and socioeconomic barrier to maternal healthcare. Based on the development, the provinces were categorized into three regions: Java–Bali, more-developed other islands and less-developed other islands [17]. We also included the type of locations (urban or rural area) as our contextual variable.

We also calculated an aggregate measure to represents the socioeconomic barrier at the community level: the proportion of poor women living in the community (% poor women). The aggregate measure was calculated from all women in our IDHS 2012 sample (*n* = 13,215, weighted *n* = 13,484).

### Statistical analysis

All analyses were conducted in the Statistical Analysis System (SAS) software version 9.4 [18]. To estimate the burden of maternal morbidity, we calculated the weighted crude proportions of self-reported morbidity. Because PROM was only included in IDHS 2007 and 2012, we also reported the labor and maternal morbidity proportions by excluding PROM from IDHS 2007 and IDHS 2012 data. Thus, we obtained more comparable estimates between the three waves of IDHS. Socioeconomic status (SES) (wealth and education level) and knowledge on pregnancy morbidity might influence self-report of maternal morbidity. Therefore, we also estimated the adjusted predicted probability of maternal morbidity controlling for these variables. We conducted multivariable logistic regressions with knowledge and SES as covariates. The predicted probabilities from these logistic regressions were used to calculate the adjusted estimates of maternal morbidity. These adjusted estimates can reduce bias due to differences in self-reporting by knowledge and SES between the three IDHS waves.

We used Chi-square to assess the differences in maternal morbidity and missed opportunity proportions. We used ANOVA to evaluate the differences in means of continuous variables (age, parity and live births). All analyses were weighted to take account the sampling scheme.

To estimate the determinants of missed opportunities, we used multilevel mixed-effects logistic regressions. The contextual variables included geographic location (region and area) and % poor women living in the community. The individual-level variables included women’s socioeconomic and demographic characteristics. The adjusted odds ratio (aOR) of fixed-effects reflected the likelihood of missed opportunity in the women. The Intra Class Correlation (ICC) reflected the proportion of variance explained by variances in the community level. Cross-level interactions were tested and retained if they were statistically significant at *p *< 0.05.

## Results

### Burden of maternal morbidity

For maternal morbidity, we analyzed data from 41,592 women (weighted *n* = 40,039) and 49,493 live births (weighted *n* = 46,340). Table 1 shows the women’s sociodemographic characteristics in the three waves of IDHS. There was an increase of mean age at delivery from 29.12 years in 2002 to 30 years in 2012 (*p* < 0.01). Meanwhile, there was a statistically significant decrease of mean parity (Table 1). There was a statistically significant increase in the proportion of women attending college or higher education from 6.0% in 2002 to 12.9% in 2012. The proportion of women who knew about the danger in pregnancy has also increased significantly, from 40.6% in IDHS 2002 to 61.1% in IDHS 2012. Information on health insurance membership was only available in IDHS 2012. Approximately 37.6% of women had insurance membership, either through social, employee or private insurance schemes.

**Table 1.** Weighted proportion of women’s sociodemographics characteristics.

Characteristics (weighted %, SD)	IDHS		
	2002	2007	2012
Live born	15,834	18,437	15,222
(weighted live born)	14,778	16,358	15,203
Total number of women	13,128	15,249	13,215
Weighted number of women	12,580	13,975	13,484
**Age** (mean, SD)*	29.12 (6.32)	29.71 (6.25)	30.00 (6.52)
**Parity** (mean, SD) *	2.52 (1.74)	2.42 (1.55)	2.15 (1.38)
**Live births last five years** (mean, SD)*	1.18 (0.40)	1.17 (0.40)	1.13 (0.37)
Wealth (weighted %, SD)*			
Richest	18.3 (0.5)	18.7 (0.5)	20.9 (0.5)
Richer	19.0 (0.6)	19.0 (0.5)	22.2 (0.5)
Middle	19.8 (0.6)	19.7 (0.5)	20.5 (0.5)
Poorer	19.3 (0.5)	19.6 (0.5)	19.3 (0.5)
Poorest	23.6 (0.5)	23.0 (0.4)	17.1 (0.4)
**Have insurance (weighted %, SD)**	n/a	n/a	37.6 (0.6)
Education (weighted %, SD)*			
College and higher	6.2 (0.3)	8.0 (0.3)	13.7 (0.4)
Secondary school	40.9 (0.7)	47.8 (0.6)	55.9 (0.6)
Primary school or lower	52.9 (0.7)	44.3 (0.6)	30.4 (0.6)
Region (weighted %, SD)*			
Java–Bali	56.7 0.6)	54.5 (0.5)	57.9 (0.5)
Other islands – more developed	30.6 (0.5)	30.5 (0.4)	28.1 (0.4)
Other islands – less developed	12.7 (0.3)	15.0 (0.3)	14.0 (0.3)
Area (weighted %, SD)*			
Urban	46.7 (0.7)	41.6 (0.6)	53.3 (0.6)
Rural	53.3 (0.7)	58.4 (0.6)	46.7 (0.6)
Distance to healthcare*			
Big problem	13.4 (0.4)	16.6 (0.4)	8.9 (0.3)
Not a big problem	86.6 (0.4)	83.4 (0.4)	91.1 (0.3)
Know danger in pregnancy (weighted %, SD)*	40.6 (0.7)	48.2 (0.6)	61.2 (0.6)

* *p*-value for differences between the three IDHS waves were significant at *p* < 0.05.

The crude proportion of self-reported maternal morbidity significantly increased from 38.3% in 2002 to 50.5% in 2007 and 53.7% in 2012. Most of these women (> 90%) reported morbidity during labor. Moreover, more than 10% of these women experienced morbidities during both pregnancy and labor (Table 2). When we excluded PROM from the analyses, the results still showed a significant increase of maternal and labor morbidity proportions (Table 2).

**Table 2.** Weighted proportions of self-reported morbidities among live births.

Period of morbidity (weighted %, SD)	IDHS		
	2002	2007	2012
Live born	15,834	18,437	15,222
(weighted live born)	14,778	16,358	15,203
**Crude estimates^a^**			
Crude maternal morbidity^a^	38.3 (0.6)	50.5 (0.6)	53.7 (0.6)
Crude pregnancy morbidity^a^	7.2 (0.3)	10.8 (0.4)	13.2 (0.4)
Crude labor morbidity^a^	35.4 (0.6)	46.7 (0.6)	49.4 (0.6)
Both pregnancy and labor morbidity^a^	4.2 (0.3)	7.0 (0.3)	8.9 (0.3)
**Estimates without PROM^b^**			
Maternal morbidity^b^	38.3 (0.6)	47.9 (0.6)	50.1 (0.6)
Labor morbidity^b^	35.4 (0.6)	43.6 (0.6)	45.1 (0.6)
Both pregnancy and labor morbidity^b^	4.2 (0.3)	6.5 (0.3)	8.2 (0.3)

All comparisons of weighted morbidity proportion between the three IDHS waves were significant at *p* < 0.05.

^a^ Crude labor morbidity from self-reported data of IDHS.

^b^ Maternal and labor morbidity with exclusion of PROM for 2007 and 2012 (PROM was recorded only in 2007 and 2012).

Our adjusted predicted probability also showed a significant increase in morbidity throughout the three waves of IDHS. Pregnancy complications were reported by an estimated 9.3% of women in 2002, 10.2% of women in 2007 and 12.6% women in 2012 (*p* < 0.01). Labor complications were reported by an estimated 42.4% of women in 2002, 43.6% of women in 2007 and 46.4% of women in 2012 (*p* < 0.01). The estimated proportion of women who reported maternal morbidity also increased from 45.2% in 2002 to 47.1% in 2007 and 51.4% in 2012 (*p* < 0.01).

We also evaluated the proportion of maternal morbidity types. There were no significant differences in the proportion of morbidity types between the three IDHS. In 2012, the most frequently reported morbidity during pregnancy were classified as others (53.4%). These conditions included hypertension, dizziness, breech position of the fetus and swelling. The second-highest morbidity during pregnancy was bleeding (25.0%), followed by prematurity (14.9%), fever (4.7%) and convulsion (2.1%). During delivery, the most frequently reported morbidity was prolonged labor (49.1%). This was followed by premature rupture of membrane (21.1%), bleeding (10.6%), infection (10.6%), convulsion (2.3%) and other (6.4%).

### Missed opportunities in maternal morbidity

For analysis of healthcare utilization, we analyzed data on women with maternal morbidity (*n* = 19,556). In all healthcare indicators, the rate of non-utilization has decreased from 2002 to 2012 (Table 3). A steeper decrease was observed in SBA non-utilization, with almost 75% relative reductions from 2002 to 2012. Delivery outside a healthcare facility had also decreased significantly, with 55% relative reduction. In IDHS 2012, the proportion of births outside health facilities that were helped by SBA was 57.8% (data not shown).

**Table 3.** Weighted proportion of non-utilization of maternal healthcare for women experiencing maternal morbidity during the last pregnancy.

Healthcare non-utilization (weighted %, SD)	IDHS		
	2002	2007	2012
*N*	5089	7705	6762
Weighted *n*	4855	7070	7301
No WHO standard ANC^a^	30.0 (1.1)	27.4 (0.8)	20.0 (0.7)
No SBA^b^	27.6 (1.0)	19.5 (0.7)	7.1 (0.5)
Delivery outside of health facility^c^	55.6 (1.1)	46.2 (0.9)	25.0 (0.7)
No SBA and delivered outside of health facility^d^	27.6 (1.0)	19.3 (0.7)	6.9 (0.4)

All comparisons of healthcare non-utilization between the three IDHS waves were significant at *p* < 0.01.

^a^ Missing data on ANC (overall) = 1252(6.4%); missing data on ANC (by IDHS) = 395(7.8%) in 2002, 635(8.2%) in 2007, and 222(3.7%) in 2012.

^b^ Missing data on SBA (overall) = 67 (0.3%); %); missing data on SBA (by IDHS) = 10 (0.2%) in 2002, 44 (0.6%) in 2007, and 13(0.2%) in 2012.

^c^ Missing data on facility birth/FB (overall) = 10 (0.05%); missing data on FB (by IDHS) = 0 (0%) in 2002, 8 (0.04%) in 2007, and 2 (0.03%) in 2012.

^d^ Missing data on SBA and FB (overall) = 74 (0.4%); missing data on SBA and FB (by IDHS) = 10(0.2%) in 2002, 49 (0.6%) in 2007, and 15 (0.2%) in 2012.

Table 4 shows the analyses on sociodemographic determinants in IDHS 2012 (*n* = 6762, weighted *n* = 7301). For all three maternal healthcare indicators, the highest proportion of under-utilization happened in women younger and multiparous women. Socioeconomic and geographic factors remain the major determinants for healthcare non-utilization. Women of low SES had higher proportions of non-utilization compared to women of higher SES. We also observed differences by geographic location. In general, women from low-resource settings had lower utilization of maternal healthcare compared to women from other areas.

**Table 4.** Weighted proportion and multilevel logistics regression of sociodemographic factors related with maternal healthcare non-utilization, IDHS 2012.

Sociodemographics Determinants	No WHO standard ANC			No SBA			No FB		
	*n*	Weighted % (SD)	aOR (95%CI)	*n*	Weighted % (SD)	aOR (95%CI)	*n*	Weighted % (SD)	aOR (95%CI)
Total women	6762	20.0 (0.7)	–	6762	7.1 (0.5)	–	6762	25.0 (0.7)	–
**Age (years)**									
< 20	214	34.2 (4.7)	2.5 (1.8–3.3)	223	12.4 (3.3)	1.5 (1.0–2.4)	223	31.7 (4.3)	1.2 (0.8–1.6)
20–35	4986	18.5 (0.8)	Ref	5133	6.8 (0.5)	Ref	5141	24.5 (0.8)	Ref
> 35	1340	21.2 (1.5)	0.9 (0.8–1.1)	1393	7.4 (1.0)	0.8 (0.6–1.1)	1396	25.8 (1.6)	0.8 (0.7–1.0)
**Parity**									
1	2660	16.7 (1.0)	Ref	2726	5.4 (0.6)	Ref	2728	21.2 (1.0)	Ref
2	1927	17.7 (1.2)	1.2 (1.0–1.4)	1973	7.0 (0.8)	1.3 (1.0–1.6)	1977	22.6 (1.3)	1.1 (1.0–1.3)
>2	1953	26.4 (1.4)	1.7 (1.4–2.0)	2050	9.9 (0.9)	1.3 (1.0–1.8)	2055	33.9 (1.5)	1.5 (1.2–1.8)
**Wealth**									
Richest	1166	11.5 (1.3)	Ref	1173	1.4 (0.5)	Ref	1176	8.5 (1.1)	Ref
Richer	1367	16.6 (1.4)	1.3 (1.0–1.6)	1388	3.9 (0.8)	1.1 (0.9–1.4)	1388	17.4 (1.4)	1.6 (1.3–2.0)
Middle	1335	19.5 (1.5)	1.5 (1.2–1.8)	1380	5.8 (1.0)	2.5 (1.5–4.1)	1381	25.9 (1.7)	2.1 (1.7–2.7)
Poorer	1345	21.3 (1.5)	1.5 (1.2–1.9)	1395	8.6 (1.1)	3.5 (2.1–5.9)	1397	29.3 (1.6)	2.1 (1.6–2.6)
Poorest	1327	33.9 (1.8)	2.5 (1.9–3.2)	1413	19.5 (1.5)	7.6 (4.6–12.8)	1418	52.5 (1.9)	3.8 (3.0–5.0)
**Insurance**									
Yes	3891	16.5 (1.0)	Ref	4028	6.6 (0.7)	Ref	4033	23.2 (1.1)	Ref
No	2644	21.3 (0.9)	1.4 (1.2–1.6)	2716	7.4 (0.6)	1.1 (0.9–1.4)	2722	26.0 (0.9)	1.1 (0.9–1.2)
**Education**									
College and higher	976	11.0 (1.3)	Ref	985	0.9 (0.4)	Ref	986	8.4 (1.0)	Ref
Secondary school	3836	18.2 (0.8)	1.3 (1.1–1.7)	3934	4.4 (0.5)	2.6 (1.3–5.2)	3939	21.2 (0.9)	2.0 (1.5–2.6)
Primary school or lower	1728	26.1 (1.4)	1.7 (1.3–2.2)	1830	15.2 (1.2)	5.7 (2.8–11.8)	1835	39.7 (1.6)	3.5 (2.6–4.7)
**Region**									
Java–Bali	2162	15.8 (1.0)	Ref	2196	6.7 (0.7)	Ref	2199	18.1 (1.0)	Ref
Other more-developed	2511	23.9 (1.0)	1.5 (1.1–1.9)	2590	6.0 (0.5)	0.7 (0.4–1.2)	2596	34.4 (1.1)	2.1 (1.2–3.7)
Other less-developed	1867	30.1 (1.2)	2.1 (1.6–2.8)	1963	11.2 (0.8)	1.4 (0.8–2.4)	1965	40.6 (1.1)	3.4 (1.9–5.9)
**Area**									
Urban	3532	17.2 (0.9)	Ref	3599	4.1 (0.5)	Ref	3604	15.0 (0.9)	Ref
Rural	3008	22.4 (1.0)	0.9 (0.7–1.2)	3150	10.7 (0.7)	1.9 (1.2–3.1)	3156	63.1 (1.2)	2.6 (1.6–4.1)
**% poor women in community**									
< 20% *	2233	16.3 (0.9)	Ref	2284	2.5 (0.4)	Ref	2287	12.3 (0.8)	Ref
≥ 20% *	4307	20.2 (0.8)	1.2 (0.8–1.6)	4465	8.0 (0.5)	1.4 (0.8–2.7)	4473	27.5 (0.8)	1.4 (0.8–2.3)
**Random effects**									
ICC unadjusted (%)			7.0			24.2			30.9
ICC adjusted (%)			2.1			9.7			13.8

All comparisons of the weighted proportion in Table 4 were significant at p < 0.05.

aOR = Adjusted Odds Ratio, CI = Confidence Interval.

ICC = Intra Class Coefficient

ICC adjusted = obtained from model with all covariates.

ICC unadjusted = obtained from unconditional model.

*Showing the frequency of women who lived in the community with the defined contextual characteristics, and their proportion of non-utilization.

Missing data on ANC = 222 (3.7%).

Missing data on SBA = 13 (0.2%).Missing data on facility birth = 2 (0.03%).

In the multilevel regressions, we found no significant cross-level interactions; therefore, the interaction terms were dropped from the analyses. For WHO standard ANC, SES was the strongest predictor for utilization. Women from the poorest family had highest odds for non-utilization of ANC (aOR 2.5, 95%CI 1.9–3.2) compared to women from the richest family. Younger and multiparous women were at higher risk for non-utilization (Table 4). Not having health insurance increased the likelihood of not having ANC (aOR 1.4, 95% CI 1.2–1.6). At the community level, there were geographic disparities for ANC non-utilization. Women from the less-developed region had the highest risk for non-utilization compared to women living in other areas. However, there were no significant differences between urban and rural areas (aOR 0.9, 95%CI 0.8–1.6). After adjustment for fixed effects, the proportion of ANC non-utilization explained by the community characteristics was quite small (adjusted ICC = 2.1%). Community-level poverty was not a significant predictor for ANC (Table 4).

Despite the relatively low non-utilization of SBA, significant differences by sociodemographic factors persisted (Table 4). In the adjusted multilevel logistic regression, the main predictor for non-utilization were socioeconomic factors and area of residence. Compared to ANC or facility birth, the highest socioeconomic disparity was observed in SBA. The proportion of non-utilization in the poorest women was almost 16 times compared to the richest women (aOR 7.6, 95%CI 4.6–12.8). Health insurance membership, age and parity were not significant predictors for missed opportunity in SBA. At the community level, living in rural areas increased the likelihood for missed opportunities in SBA (aOR 1.9, 95%CI 1.2–3.1). However, region was not a significant predictor for SBA non-utilization. A larger adjusted ICC was observed for SBA (9.7%) compared to ANC.

Multiparous women were more likely to have non-institutional births. SES (wealth and education) was the strongest predictor for facility birth. Women of low SES were most likely to deliver outside of health facility (Table 4). Interestingly, health insurance was not a significant predictor for non-institutional births (aOR 1.1, 95%CI 0.9–1.2). At the community level, regional disparities were most evident in facility birth compared to ANC and SBA. Women from the less-developed region were more likely to deliver outside of health facility (Table 4). Living in rural areas also increased the likelihood of non-institutional delivery (aOR 2.6, 95%CI 1.6–4.1). In addition, the adjusted ICC for facility birth were 13.8%, the highest compared to ANC and SBA.

## Discussion

### Burden of maternal morbidity

Our study showed a relatively high prevalence of self-reported maternal morbidity in Indonesia. Almost half of the women experienced pregnancy and/or labor morbidity, with increasing trends from 2002 to 2012. Although attenuated, we still observed significant increases in maternal morbidity when we excluded PROM. Additionally, the adjusted estimation of maternal morbidity also indicated significant increases in the morbidities. The most prominent increase happened in labor morbidity. The significant increases of crude and estimated proportion signifies the importance of not only improving self-awareness to maternal morbidity but also improving the coverage and the quality of maternal healthcare [19–22]. Previous studies reported that 1.7–2.0% of women had severe maternal complications [23,24], while 50.1–53.0% of women had non-life threatening maternal morbidity [25]. With the relatively high burden of morbidity in Indonesia as well as these developing countries, it is crucial to reconcile the scope of maternal health programs to include maternal morbidity. Specifically, to improve the detection of a high-risk population, early case identification and prompt management of maternal morbidity.

In IDHS 2012, most pregnancy morbidities were related to predisposing as well as pregnancy-related conditions. These conditions included hypertension, fetal position, dizziness and swelling. Further investigation to classify these different types of morbidity is crucial to design more detailed intervention on pregnancy morbidity. Meanwhile, prolonged labor was the most prevalent labor morbidity, followed by PROM and infection. Previously, a retrospective hospital-based study reported hemorrhage and pregnancy-related hypertension as the two major causes of maternal near-miss in Indonesia [14]. The observed differences between these two studies might derive from the different baseline population. Additionally, there might be different determinants of the community-level vs. hospital-based maternal morbidity [26]. Most previous studies on risk factors of maternal morbidity focused on severe morbidities or near miss. These studies have identified several important risk factors for severe maternal morbidity including younger [24,27,28] or older age [24,27–30], parity [30,31], history of complications in previous pregnancy [24,29–31], poverty [31,32] and less contact with healthcare provider [31–33]. Therefore, studies evaluating sociodemographics and biologic determinants of non-life threatening maternal morbidities are necessary. Particularly, to provide more comprehensive information on the risk factors of the maternal morbidity continuum.

### Missed opportunities in maternal morbidity

One important strategy in improving maternal health is by increasing the coverage and quality of maternal healthcare [34,35]. In 2012, the rates of non-utilization in women with morbidity were relatively lower than general Indonesian women [13]. However, it is still alarming that among women with maternal morbidity, there was a relatively high non-utilization of healthcare; specifically, for WHO standard ANC and facility delivery. Intervention to address this missed opportunity is important. Specifically, to reduce the high proportion of delivery outside a health facility. This intervention is crucial because, during labor emergency, distance to healthcare and the different quality of care can cause unnecessary consequences for the woman and/or the newborn [36].

Our study showed that despite having similar medical needs due to their morbidities, differences by socioeconomic and geographic conditions persisted. Previous studies that have also indicated these disparities did not specifically address women with maternal morbidity [37–39]. Our multilevel analyses showed the importance of community level factors, specifically geographical location. The community characteristics were most important for missed opportunities in institutional delivery, compared to ANC and SBA. Living in low-resource population (less-developed region or rural area) increased the likelihood of delivery outside of health facility. This condition might suggest the importance of addressing geographic barrier for the more urgent nature of the delivery process. Meanwhile, for the relatively less-urgent ANC, socioeconomic factors were the stronger determinants for non-utilization. However, our data suggested the presence of socioeconomic disparity in all three healthcare indicators, although more evident in SBA and facility birth. These geographic and socioeconomic disparities remain to be one of the main challenges for healthcare distribution in Indonesia [39–41].

Our analysis showed that insurance membership improved WHO standard ANC. However, we failed to show the significance of insurance membership in reducing non-utilization of SBA and facility birth. Previous findings suggested that health insurance was more beneficial for low SES women [42–44]. Further analysis to assess whether this effect persisted in women with higher SES, specifically those with medical needs, will be important for policy recommendation. We also demonstrated the need to address low utilization among younger and multiparous women. This finding is crucial because these women are at higher risk for morbidity, including those leading to near-miss or even mortality [31].

To reduce these missed opportunities, it is important for the government to implement multiple levels of intervention. In addition to the wide population-based strategies [45–47], health system strengthening and high-risk approach strategies are also important in Indonesia. The high-risk intervention can be conducted through home visits and/or counseling, particularly for vulnerable women [48,49]. It is also important to extend the social health insurance scheme, particularly, for low-SES women to reduce financial barrier. A previous study on health insurance coverage in Indonesia showed that the low-SES women had less insurance coverage compared to richer women. Moreover, the majority of health insurance in this disadvantaged population was obtained through pro-poor social insurance scheme [50]. In addition, advancing the distribution of healthcare to the less developed region is also important to address the geographic barrier for institutional birth.

### Limitations and strengths

Due to the nature of self-reported morbidities, this study is susceptible to biases: first, information bias due to differential reporting by SES and knowledge on maternal morbidity. In this paper, we estimated the adjusted predicted probability of maternal morbidity. However, even after the adjustment, residual confounding might persist and influence our estimates of maternal morbidity proportion. Despite the limitation of self-reporting, a validation study showed that self-reported maternal morbidity has relatively good sensitivity and specificity [51]. Second, survival bias. There were women who experience morbidity but died, which we could not assess in this study. Third, recall bias due to the retrospective self-reported morbidity. To attenuate this bias, we only included information from the most recent birth in the analyses for missed opportunities. However, we still included all information for maternal morbidities. Another limitation of this study was the inability to assess the severity of maternal morbidity, early-pregnancy morbidity (i.e. abortion, ectopic pregnancy), or postpartum morbidity.

However, this paper has important strengths and contributions. This study describes and specifies the community-level burden of maternal morbidity in Indonesia. This study also presents the extent of missed opportunities as well as identifying vulnerable populations for healthcare non-utilization. The socioeconomic and geographic disparities that we observed in our study provide more meaningful insights because they occurred in women with morbidity. All of these women ideally should receive healthcare. These results can be the basis in tailoring more applicable public health interventions. Additionally, this study signifies the importance of analyzing available maternal morbidity data, specifically in low–middle-income countries [27]. Our study also demonstrated the urgency for broadening the scope of maternal health programs to include maternal morbidities.

## Conclusion

Indonesia has a relatively high maternal morbidity prevalence. We found that almost half of Indonesian women reported morbidity during the maternal period. Furthermore, the prevalence of pregnancy and/or labor morbidity is increasing throughout the study period. These findings indicate the importance of expanding the scope of the maternal health programs to include maternal morbidity. With limited information on determinants of non-life threatening maternal morbidity, identifying the high-risk population is crucial to develop more effective intervention.

Missed opportunities for women with maternal morbidity are also concerning; specifically, the relatively high under-utilization and sociodemographics disparities of WHO standard ANC and facility birth. For all indicators, the under-utilization was more prevalent in younger and multiparous women. Moreover, women of low SES and women who lived in low-resource settings were more likely to not utilize maternal healthcare. Further research should focus on designing appropriate intervention to improve utilization in these vulnerable population. In particular, by implementing high-risk intervention strategy for younger, multiparous women of low SES who live in disadvantaged regions in Indonesia.

## Paper context

In developing countries, maternal health interventions have been more focused on mortality, with limited data on maternal morbidity. We addressed the information gap in Indonesia by showing the high prevalence of community-level maternal morbidity, specifically during labor. Additionally, we demonstrated the presence of socioeconomic and geographic disparities in maternal healthcare utilization among women with maternal morbidity. Further efforts should focus on identifying risk factors for maternal morbidity and improving healthcare utilization for vulnerable population.
